# Highly Stretchable Sound‐in‐Display Electronics Based on Strain‐Insensitive Metallic Nanonetworks

**DOI:** 10.1002/advs.202001647

**Published:** 2020-11-23

**Authors:** Seungse Cho, Dong‐hee Kang, Hyejin Lee, Minsoo P. Kim, Saewon Kang, Ravi Shanker, Hyunhyub Ko

**Affiliations:** ^1^ School of Energy and Chemical Engineering Ulsan National Institute of Science and Technology (UNIST) Ulsan Metropolitan City 689‐798 Republic of Korea

**Keywords:** electroluminescent loudspeakers, sound‐in‐display electronics, strain‐insensitive silver nanowire electrodes, stretchability

## Abstract

The growing importance of human–machine interfaces and the rapid expansion of the internet of things (IoT) have inspired the integration of displays with sound generation systems to afford stretchable sound‐in‐display devices and thus establish human‐to‐machine connections via auditory system visualization. Herein, the synchronized generation of sound and color is demonstrated for a stretchable sound‐in‐display device with electrodes of strain‐insensitive silver nanowires (AgNWs) and emissive layers of field‐induced inorganic electroluminescent (EL) phosphors. In this device, EL phosphors embedded in a dielectric elastomer actuator (DEA) emit light under alternating‐current bias, while audible sound waves are simultaneously generated via DEA actuation along with input sound signals. The electroluminescence and sound‐generation performances of the fabricated device are highly robust and reliable, being insensitive to stretch‐release cycling because of the presence of the AgNW stretchable electrodes. The presented principle of integrating light emission and acoustic systems in a single stretchable device can be further expanded to realize sound‐in‐display electronics for IoT and human–machine interface applications.

## Graphical abstract

A stretchable sound‐in‐display with electrodes of strain‐insensitive silver nanowires and emissive layers of field‐induced inorganic electroluminescent (EL) phosphors are proposed for generation of synchronized acoustic and visual information. The loudspeaker simultaneously emits light by the EL and generates sound by the vibration of the dielectric elastomer actuator under the same bias, allowing the synesthetic perception of both color and sound.

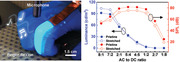

## 1 Introduction

The growing demand for bezel‐less,^[^ ^1^ ^]^ interactive,^[^ ^2^ ^]^ and smart^[^ ^3^, ^4^ ^]^ displays, driven by the need to maximize user experience and the user‐display interface, has inspired the integration of diverse functions (e.g., speakers, sensors, actuators, and cameras) into or under displays. For example, interactive displays with integrated sensors visualize various external stimuli such as touch,^[^ ^5^, ^6^, ^7^ ^]^ strain,^[^ ^8^, ^9^, ^10^ ^]^ and pressure^[^ ^11^, ^12^, ^13^, ^14^ ^]^ and stimulate the human sense of vision to achieve their intuitive and instant perception. Prior research has mostly focused on the visualization of mechanical stimuli for tactile display and human–machine interface applications, leaving the visualization of sound waves underexplored. When visual stimuli are accompanied by acoustic ones or vice versa, they are easily perceived by humans via the auditory–visual synesthesia because of the synergistic effect of conscious and reliable perceptions.^[^ ^15^, ^16^ ^]^ Similarly, the integration of a loudspeaker and a display in a single device is expected to enable the generation of synchronized acoustic and visual information and thus allow the synesthetic perception of information and maximized user experience. Furthermore, sound‐in‐display technologies can be combined with stretchable and thin film form factors to afford stretchable sound‐in‐display solutions that can be widely applied in the internet of things (IoT), wearable devices including visible assistance for people with hearing problems, medical devices using sound‐to‐vision conversion, and human–machine interfaces attached on arbitrary curvilinear objects and body parts.

Although sound‐emitting thin film electroluminescent (EL) devices can be fabricated using piezoelectric ceramics such as barium titanate,^[^ ^17^ ^]^ Pb(Mg_1/3_Nb_2/3_)O_3−_ *_x_*PbTiO_3_,^[^ ^18^ ^]^ and lead zirconate titanate^[^ ^19^ ^]^ with zinc sulfide‐based phosphors as emitting layers, the applications of these devices in stretchable electronics are limited by the complicated fabrication processes requiring high temperatures, hard substrates, and rigid ceramic insulators. Kim et al.^[^ ^20^ ^]^ demonstrated the simultaneous generation of color and sound in a soft photonic‐crystal device with electro‐actuation capability. However, even though this photonic skin showed the dual functionality of color tuning and sound generation in response to both direct‐current (DC) and alternating‐current (AC) biases, its practical usability in low‐power and stretchable electronics was limited by the need for very high operating voltages (several kV) and the lack of stretchability. More recently, Kim et al.^[^ ^21^ ^]^ have presented flexible artificial synesthesia electronic devices that employ inorganic EL phosphors to realize light emission and use piezoelectric polymer films to visualize input sound signals and generate sound. Despite the prominent brightness and sound performances of these thin film‐based devices, their applications in stretchable electronics are limited by the inelasticity of conducting electrodes and piezoelectric materials. To have a wide application scope, sound‐generating displays should exhibit both bending capability and stretchability, that is, should withstand large mechanical deformations and conformably wrap around arbitrary curvilinear surfaces and human bodies.^[^ ^22^, ^23^, ^24^ ^]^

Stretchable AC electroluminescent (ACEL) devices have attracted much attention as next‐generation light sources because of their high robustness and stable performance under large mechanical deformation, simple fabrication procedure, and high brightness.^[^ ^12^, ^25^, ^26^, ^27^, ^28^, ^29^, ^30^, ^31^ ^]^ Ionic hydrogels,^[^ ^12^, ^25^ ^]^ ionic conductors,^[^ ^27^, ^29^ ^]^ carbon nanotubes (CNTs),^[^ ^30^ ^]^ and silver nanowires (AgNWs)^[^ ^26^, ^28^ ^]^ have been widely used for the fabrication of stretchable electrodes to realize stretchable ACEL devices capable of stable operation under large strain. Among these devices, those based on hydrogels and ionic conductors show large stretchability but require overly high operating voltages because of the low conductivity of stretchable electrodes.^[^ ^12^, ^25^, ^27^, ^29^ ^]^ Conversely, stretchable ACEL devices with CNT‐ and AgNW‐based stretchable electrodes show high luminance at relatively low operating voltages but exhibit low stretchability because of the inherent limitations of conducting materials.^[^ ^26^, ^30^, ^32^ ^]^ Despite the notable progress in the fabrication of stretchable ACEL devices, no attempts have been made to construct stretchable sound‐in‐display electronics using stretchable ACEL devices for the generation of synchronized sound and vision information, which can enhance the user experience and interface in diverse human–machine interface applications.

Herein, a stretchable sound‐in‐display device generating synchronized acoustic and visual information is fabricated using a stretchable EL loudspeaker with an emissive layer of copper‐doped zinc sulfide (ZnS:Cu) and a dielectric elastomer actuator (DEA) matrix that are sandwiched between stretchable electrodes. When an AC bias is applied to this loudspeaker, EL occurs at the emissive layer, and an audible sound wave is generated through DEA matrix vibration. The intensities of light emission and output sound depend on the frequency and amplitude of the applied bias. In particular, light emission and sound‐generation performances are invariant under significant tensile strain because of the stable electrical connection provided by strain‐insensitive stretchable AgNW electrodes. As a result, the developed stretchable sound‐in‐display device synchronously generates light and sound even under the conditions of significant stretching.

## 2 Results and Discussion

**Figure 1:**
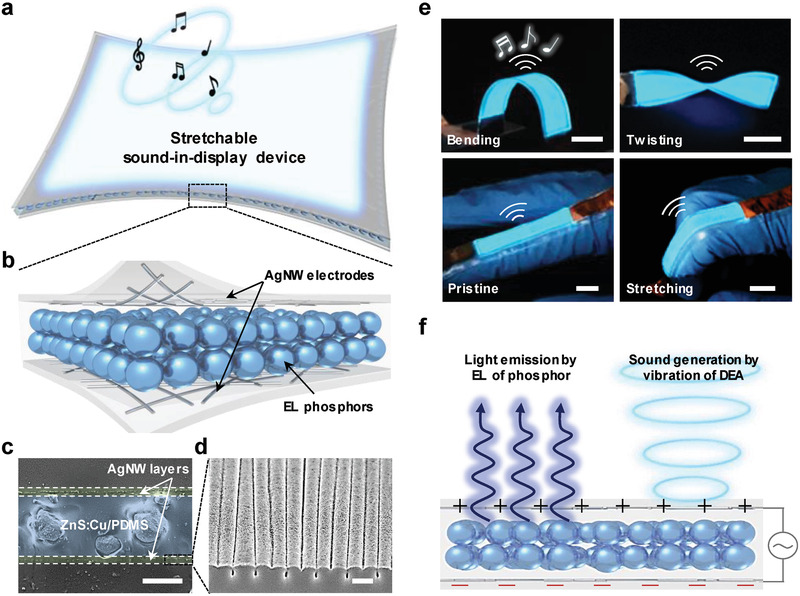
Stretchable sound‐in‐display electronics. a) Concept of a stretchable sound‐in‐display device and b) schematic image showing device geometry. c) Cross‐sectional SEM image of the device. Scale bar is 20 µm. d) SEM image of stretchable AgNW electrode. Scale bar is 5 µm. e) Operation of the sound‐in‐display device with sound‐synchronized EL under bending, twisting, and stretching on the index finger. An audible sound and blue luminescence are generated (AC 60 V and 5 kHz). All scale bars are 1.5 cm. f) Schematic operation mechanism of the sound‐in‐display device.

The concept of integrating a color module and a speaker in a single stretchable unit to afford a so‐called stretchable sound‐in‐display device capable of light/sound synchronization is illustrated in **Figure** **1**a. Figure 1b,c show the schematic structure and a cross‐sectional scanning electron microscopy (SEM) image of a stretchable sound‐in‐display device consisting of a ZnS:Cu/ polydimethylsiloxane (PDMS) EL layer sandwiched between stretchable AgNW electrodes on PDMS layers. The homogeneous size distribution (average size ≈18.2 µm; Figure S1, Supporting Information) of ZnS:Cu particles and their good dispersion in the PDMS matrix layer resulted in uniform light emission without device failure under high electric fields. The stretchable AgNW electrodes featured a percolative network of AgNWs (average diameter = 21 ± 3 nm, average length = 22 ± 5 µm; Figure S2, Supporting Information) conformally coated on the wrinkled PDMS matrix (Figure 1d), which enabled the maintenance of good electrical conductivity during mechanical deformation. Figure S3, Supporting Information, shows a photograph of the semi‐transparent stretchable EL loudspeaker that simultaneously generates sound (via vibration of the ZnS:Cu/PDMS composite DEA in the emitting layer) and light (via the field‐induced EL of inorganic phosphors) under AC biases. Specifically, the loudspeaker emitted bright blue light and audible sound from both sides of the display under the application of a square pulse function (60 V, 5 kHz) even when subjected to mechanical deformations such as bending, twisting, and stretching during finger flexion (Figure 1e). DEA actuation resulted from the consecutive contraction and expansion of the DEA film in the thickness and lateral directions under the AC bias input for light emission, enabling the synesthetic perception of both color and sound (Figure 1f). Although single‐functionality devices, that is, those capable of light emission or sound generation, have been reported, the concept of stretchable sound‐in‐display electronics employing a stretchable EL loudspeaker for the generation of synchronized sound and visual information has not yet been explored (Table S1, Supporting Information). In addition, our stretchable EL loudspeaker achieved high brightness (luminance ≈ 15.1 cd m^−2^ at 120 V and 1 kHz), excellent sound‐generation performance (sound pressure level (SPL) ≈ 83.3 dB at 120 V and 10 kHz), and large stretchability (up to 150%), and was therefore favorably compared with previously reported devices listed in Table S1, Supporting Information.

Stretchable electrodes are critically important for the realization of high‐performance stretchable sound‐in‐display devices. During repetitive stretching or mechanical deformation, previously reported stretchable electrodes based on graphene,^[^ ^33^ ^]^ conducting polymers,^[^ ^34^ ^]^ CNTs,^[^ ^35^ ^]^ and hybrid systems^[^ ^28^ ^]^ experience electrical conductivity degradation due to the damage of conducting networks. Attempts to solve this problem included the deposition of AgNW networks on elastomeric matrices; however, the resulting electrodes still exhibited limited stretchability.^[^ ^36^, ^37^, ^38^, ^39^, ^40^ ^]^ Herein, strain‐insensitive stretchable electrodes fabricated by the formation of AgNW networks conformally attached to a wrinkled PDMS substrate (Figure S4, Supporting Information) were shown to maintain electrical conductivity at uniaxial tensile strains of up to 150%. Partially cured PDMS (20 min at 80 °C) was stretched to 150%, released to form wrinkles, and subsequently treated with O_2_ plasma to afford a hydrophilic surface bearing hydroxyl groups^[^ ^41^ ^]^ and thus enhance surface wettability and ensure the uniform coating of AgNWs. After the spin coating of AgNW dispersions, partially cured pre‐strained PDMS was released and fully cured (2 h at 80 °C). The stiffness differences between the stiff AgNW layer and the soft bottom PDMS layer resulted in the formation of periodical wrinkles upon the release of pre‐strained PDMS (Figure S5, Supporting Information). In addition to wrinkles, cracks were formed in the direction parallel to that of the pre‐strain because of the Poisson effect, as the release of pre‐stretched PDMS resulted in its expansion in the direction transverse to that of stretching. The softness and stickiness of partially cured PDMS enabled the strong and conformal attachment of AgNW networks on the periodic wrinkles of stretchable electrodes (Figure S6a,b, Supporting Information). In addition, the low Young's modulus of partially cured PDMS minimized the formation of surface cracks after the release of pre‐stretched PDMS. On the contrary, the use of the less sticky and less elastic fully cured PDMS resulted in the poor attachment of AgNW networks on wrinkles, and protruded nanowires together with abundant cracks were observed (Figure S6c,d, Supporting Information). The conformal adhesion of AgNW networks to partially cured PDMS endowed the corresponding stretchable AgNW electrodes with an ability to maintain electrical conductivity during repeated taping tests, while in the case of electrodes fabricated on fully cured PDMS, electrical conductivity decreased after several taping tests (Figure S7, Supporting Information).

**Figure 2:**
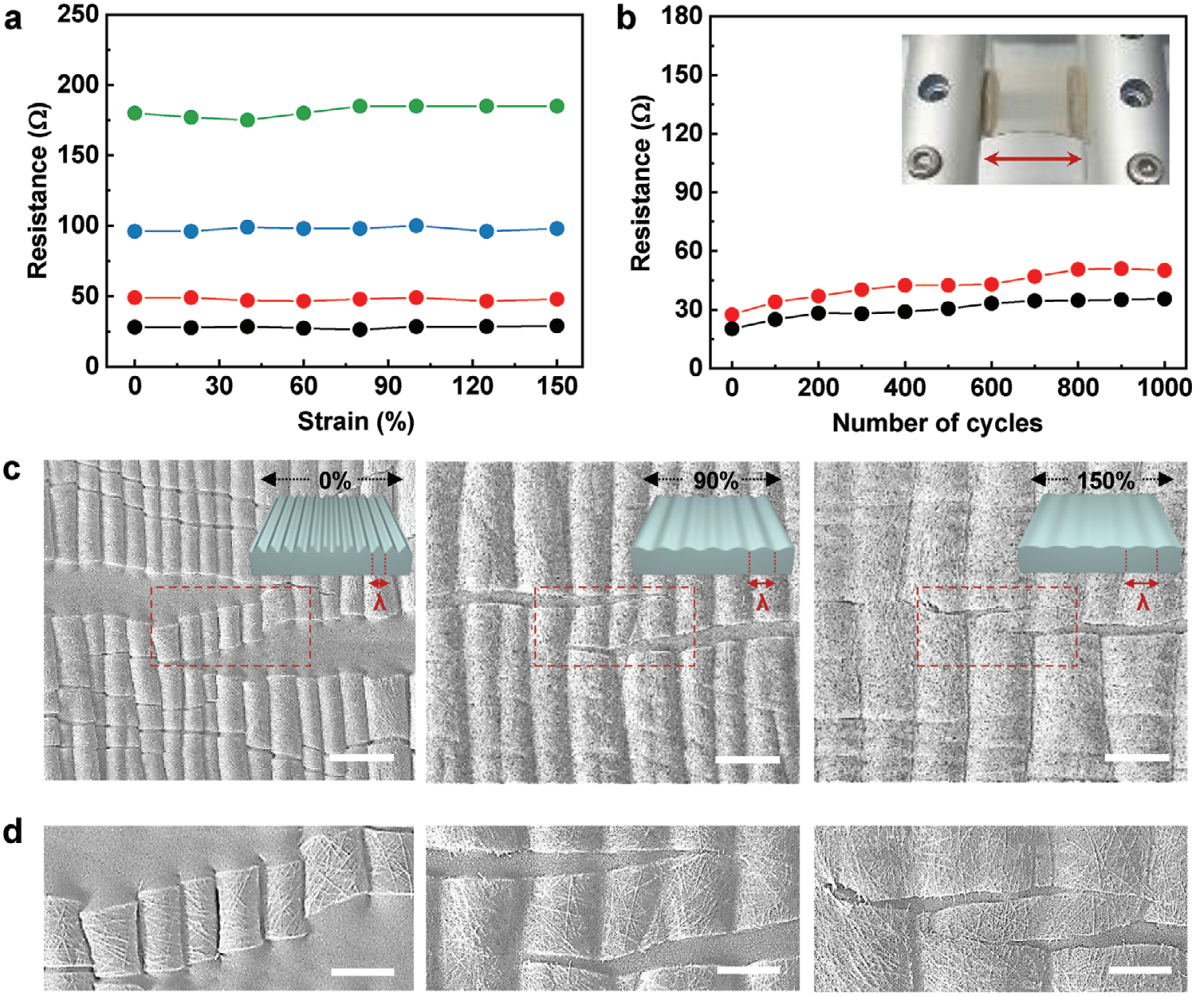
Characterization of strain‐insensitive stretchable AgNW electrodes. a) Resistance as a function of tensile strain for different initial resistances. b) Resistance evolution during repetitive stretching to 100% for 1000 cycles. Inset: photograph of the stretching system. c) SEM images of stretchable AgNW electrodes during stretching to 150%. All scale bars are 20 µm. Insets illustrate the wrinkle structures of stretched electrodes. d) Enlarged SEM images of stretchable AgNW electrodes during stretching to 150%. All scale bars are 10 µm.

The stretchable electrodes showed almost no resistance variation under uniaxial tensile straining up to a pre‐strain level of 150% regardless of their initial resistance (**Figure** **2**a), which could be easily controlled by adjusting the density of AgNW networks (Figure S8, Supporting Information). The degree of pre‐straining was a crucial parameter determining the performance of our stretchable electrodes, as their wrinkled surface could be stretched up to the applied pre‐strain level without the formation of additional cracks. The reversibility and stability of resistance observed after 1000 stretch‐release cycles for our electrodes were indicative of outstanding mechanical durability owing to the conformal attachment of AgNW networks to the wrinkled PDMS substrate, which ensures the strong adhesion of AgNW networks to the PDMS during repeated stretching/releasing (Figure 2b). On the other hand, under the above conditions, electrodes fabricated in the absence of pre‐straining showed a dramatic increase in resistance due to the failure and breakage of conducting networks (Figure S9, Supporting Information). A light‐emitting diode connected to our electrodes maintained a stable illumination intensity as the electrode was stretched to a strain of 150%, which was indicative of high electrode performance and insensitivity to strain (Figure S10, Supporting Information).

Figure 2c and Figure S11, Supporting Information, show changes in the micro‐ and macroscopic surface morphologies of stretchable AgNW electrodes during stretching to 150% strain. Stretchable electrodes can be fabricated using two different strategies based on the modification of structures or materials to be stretched.^[^ ^37^, ^42^ ^]^ The deposition of highly ductile AgNWs on wrinkled PDMS combines the advantages of these two strategies. The first strategy of using “structures that stretch” relies on the introduction of wrinkles to release the strain applied to the electrode during stretching. When tensile strain is applied to the electrode in a direction perpendicular to the wrinkle pattern, the wrinkle structures flatten to accommodate the applied strain (Figure 2c), and the wrinkle width increases with increasing tensile strain without the formation of additional cracks along parallel wrinkles. It is worth noting that the width of the original cracks formed because of the Poisson effect (Figure S5, Supporting Information) decreased with increasing tensile strain, which resulted in the generation of more conducting pathways and, hence, in stable electrical conductivity during electrode elongation (Figure 2d). The second strategy of using “materials that stretch” relies on the intrinsically high ductility of nanosized AgNWs with a large aspect ratio and stretchability.^[^ ^37^, ^43^ ^]^ Consequently, the combination of these two strategies allowed our stretchable electrodes based on AgNW networks on wrinkled PDMS to provide a stable electrical conduction pathway during stretching without notable resistance change. Moreover, no resistance changes were observed at bending radii of down to 1 mm and during repeated bending for up to 1000 cycles, which indicated the excellent electrical and mechanical durability of our stretchable electrodes (Figure S12, Supporting Information).

The sound‐in‐display concept was realized by using the EL of the inorganic phosphor for light emission and DEA vibration under the applied AC bias for sound generation (Figure 1a). In the stretchable EL loudspeaker, the ZnS:Cu phosphor/PDMS dielectric layer is sandwiched between the top and bottom AgNW electrodes. Under a sufficiently high applied voltage, trapped electrons are injected from the PDMS–ZnS:Cu interface to the phosphor conduction band and are accelerated to induce light emission via the impact ionization and excitation of luminescent dopant centers followed by their radiative relaxation (Figure S13, Supporting Information).^[^ ^44^, ^45^ ^]^ The light‐emitting performance of the constructed device was examined using a spectroradiometer in a dark box at different voltages and frequencies controlled by a function generator connected to a voltage amplifier (Figure S14, Supporting Information). The loudspeaker luminance intensity was investigated in various driving modes (sinusoidal, ramp, and square waveforms; Figure S15, Supporting Information). Notably, the EL intensity under the square pulsed bias exceeded those under sinusoidal and ramp pulsed biases, as the effective working voltage of the square waveform (0.5 *V*_pp_) exceeded those of sinusoidal (0.35 *V*_pp_) and ramp (0.29 *V*_pp_) waveforms.

**Figure 3:**
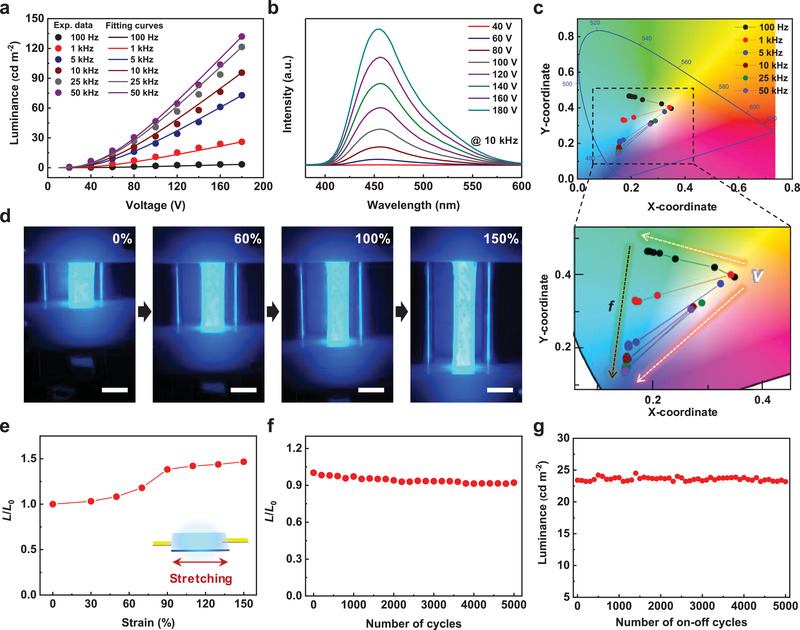
Light emission characteristics and mechanical properties of stretchable EL loudspeaker. a) Effects of voltage on the luminance of stretchable EL loudspeaker at different frequencies and the corresponding fits. b) EL spectra of stretchable EL loudspeaker recorded at different applied voltages and a fixed frequency of 10 kHz. c) Representation of color coordinates in CIE 1931 color space according to voltage‐ and frequency‐dependent emission color change. White arrows (*V*) indicate the change of color coordinates upon the increase of applied AC voltage from 20 to 180 V. Black arrow (*f*) indicates the change of color coordinates upon the increase of applied frequency from 100 Hz to 50 kHz. d) Photographs of EL loudspeaker stretched to strains of 0%, 60%, 100%, and 150% (AC 100 V, 10 kHz). All scale bars are 1.5 cm. e) Effect of stretching strain on the relative emission intensity of the stretchable EL loudspeaker. f) Stretching stability test of the stretchable EL loudspeaker under repetitive straining to 100%. g) Emission stability test of the stretchable EL loudspeaker under 5000 on–off cycles.

**Figure** **3**a shows the effects of voltage (20–180 V) and frequency (100 Hz–50 kHz) on loudspeaker luminance, revealing that light emission intensity gradually increased with increasing voltage and frequency. The relationship between luminance (*L*) and applied voltage bias (*V*) at a certain frequency is given by^[^ ^46^ ^]^

(1) $$\begin{array}{c} L=L_{0}\exp\left(-\beta /V^{0.5}\right) \end{array}$$

where *L*_0_ and *β* are device‐ and material‐dependent constants. Within the whole range of applied frequencies, the experimental data were well fitted by Equation (1) (Figure 3a and Note S1, Supporting Information). At low applied bias, the active layer acts as an insulator, becoming conductive at biases above the breakdown voltage, in which case electrons are accelerated to sufficiently high energies to excite the luminescent centers and induce a steep increase in luminance. At a frequency of 10 kHz, the luminance peak centered at 456 nm gained intensity with increasing voltage bias (Figure 3b). On the other hand, when the frequency was increased from 50 Hz to 20 kHz, the luminance peak shifted from 508 (green) to 460 nm (blue) at a fixed bias (Figure S16, Supporting Information). The above voltage‐ and frequency‐dependent emission changes can be clearly observed in the corresponding CIE 1931 chromaticity diagram (Figure 3c). As the applied bias increased from 20 to 180 V, the chromaticity coordinates shifted from the yellowish region to the green region at low frequencies, which indicated a gradual increase of illumination intensity, whereas the rapid shift from the yellowish region to the deep blue region at high frequencies indicated a steep increase of illumination intensity. The coordinate points in the yellowish region indicate insufficient illumination intensity due to low applied bias. As the frequency increased from 100 Hz to 50 kHz, the chromaticity coordinates shifted from the green region to the deep blue region. This behavior was attributed to the presence of two dopant crystallographic sites with two different types of shallow and deep traps.^[^ ^45^ ^]^ Under the condition of low frequency and weak electron/hole injection, mostly shallow traps are occupied, and green emission is observed, whereas the occupation of deeper traps at higher frequencies due to the higher‐energy injection induces a shift to blue emission. Under the same electric field strength and frequency, luminance increased with decreasing emission film thickness (Figure S17, Supporting Information) because of the larger electric field experienced by ZnS:Cu phosphors in thinner devices. Specifically, a luminance of 51.5 cd m^−2^ was achieved at a film thickness of 28.3 µm (100 V, 10 kHz).

Loudspeaker stretchability was tested by elongating the device in the uniaxial direction during EL operation (Figure 3d). Figure 3d,e show that the loudspeaker could be stably operated with bright blue emission at tensile strains of up to 150% without noticeable changes in luminance. This robust performance originated from the stable supply of applied voltage bias and current to the loudspeaker via the strain‐insensitive stretchable AgNW electrodes. As strain increased to 150%, luminance intensity also increased (Figure 3e), which was attributed to the concomitant decrease of emission layer thickness and the resulting increase in the strength of the electric field acting on the emission layer. In addition, the relative change in loudspeaker luminance after 5000 stretch‐release cycles under a strain of 100% was less than 8% (Figure 3f), indicating the superior mechanical durability and stability of the tested device. Furthermore, the loudspeaker showed a negligible change in EL intensity even after 5000 on–off cycles, which was indicative of long‐term stability (Figure 3g).

**Figure 4:**
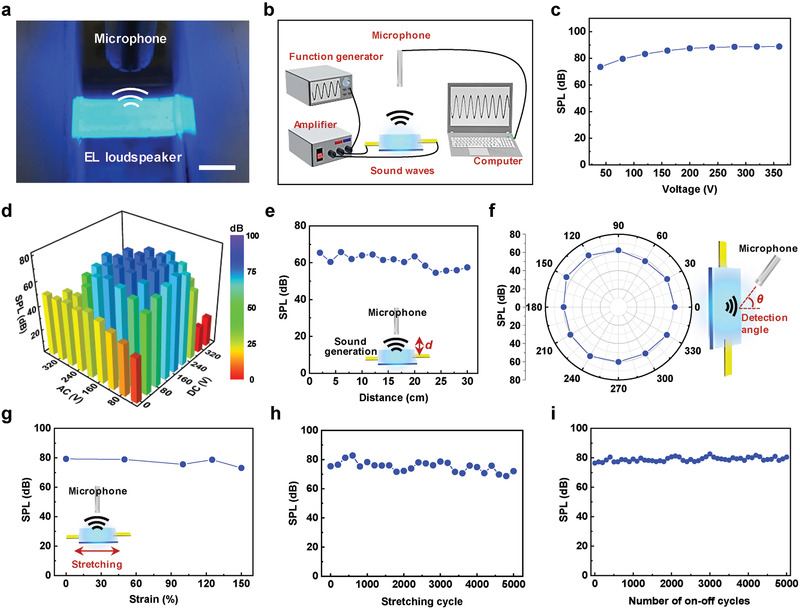
Characterization of the stretchable EL loudspeaker. a) Photograph of the stretchable EL loudspeaker during sound recording using a microphone. Scale bar is 1.5 cm. b) Acoustic measurement system in which sound emitted from the stretchable EL loudspeaker is collected by a microphone. c) Effect of applied voltage on the SPL generated by the stretchable EL loudspeaker at an AC to DC voltage ratio of 3:1. d) Sound performance of stretchable EL loudspeaker for different AC to DC voltage ratios. e) Dependence of SPL on the distance between the microphone and the stretchable EL loudspeaker. f) Effect of angle on the SPL of the stretchable EL loudspeaker at 100 V and 10 kHz. The distance between the loudspeaker and the microphone equaled 2.5 cm. g) Dependence of loudspeaker SPL on tensile strain. h) Stability of sound performance during 5000 cycles of stretching to 100% strain. i) Sound performance stability of the stretchable EL loudspeaker during repeated 5000 on–off cycles.

Field‐dependent light emission was due to the EL of ZnS:Cu phosphors, and sound waves were synchronously generated by the vibration of the DEA based on ZnS:Cu/PDMS composites under the same applied AC bias (Figure 1f). Whereas conventional speakers require complicated fabrication processes and a large volumetric speaker size for diaphragm vibration, the ZnS:Cu/PDMS DEA of our device could generate sound by converting electrical energy into the mechanical vibration of a thin film structure.^[^ ^47^, ^48^, ^49^, ^50^ ^]^ Upon the application of an electric field, attractive forces between opposite charges on the top and bottom electrodes of the DEA provide compressive forces through Maxwell stress, inducing the contraction and expansion in the thickness and lateral directions of the DEA film, respectively. **Figure** **4**a shows the stretchable EL loudspeaker generating sound in sync with the emitted light under an AC bias. Figure 4b shows the acoustic measurement system used to analyze the sound performance of the stretchable EL sound‐in‐display device inside an anechoic chamber upon the application of a voltage produced by a function generator and then amplified by an amplifier. The background noise floor inside the anechoic chamber was below 20 dB (Figure S18, Supporting Information). A commercially available microphone was used to collect and record the sound produced by the stretchable loudspeaker, and the intensity of this sound was measured under various driving modes (sinusoidal, ramp, and square waveforms; Figure S19, Supporting Information). As discussed in Figure S15, Supporting Information, the highest SPL was observed for the square pulsed bias because its effective working voltage exceeded those of sinusoidal and ramp pulsed biases.

Figure 4c shows the SPL of sound generated by the stretchable EL loudspeaker as a function of applied voltage at a frequency of 10 kHz and a AC to DC voltage ratio of 3:1. Notably, sound pressure increased with increasing applied voltage, as the degree of actuation (*S*) on a dielectric elastomer film is proportional to the square of applied voltage (*V*):^[^ ^51^ ^]^

(2) $$\begin{array}{c} S=\varepsilon_{r}\varepsilon_{0}V^{2}h^{-2}Y^{-1} \end{array}$$

where *ε*_r_ is the relative dielectric constant, *ε*_0_ is the permittivity of free space, *h* is film thickness, and *Y* is Young's modulus. DEA‐based loudspeakers are mainly operated using a combination of DC and AC voltages, similarly to electrostatic speakers.^[^ ^52^ ^]^ When the merged voltage (*V*) applied to the DEA loudspeaker is given by *V* = *B* + *A*sin(*ωt*), where *B* is the DC bias and *A* is the amplitude of the AC drive voltage, the time‐dependent actuation amplitude (*S*_AC_) in Equation (2) can be expressed as

(3) $$\begin{array}{c} S_{\mathrm{AC}}=\frac{\varepsilon_{r}\varepsilon_{0}}{h^{2}Y}\left(2BA+A^{2}\right) \end{array}$$

Equation (3) suggests that the sound performance of the stretchable EL loudspeaker depends on both DC bias and AC drive voltages. Figure 4d shows the SPL of output sound at different DC to AC voltage ratios and a frequency of 10 kHz, revealing that SPL increased as DC bias was added to the AC voltage, saturating at DC biases above 100 V. Note that the maximum combined AC‐DC voltage (*V*_max_) was limited to 400 V because of the specifications of our equipment. As sound performance is related to DEA film vibration, the acoustic response is proportional to the square of the applied voltage (as indicated in Equation (3)), which always results in the generation of a harmonic distortion (Note S2, Supporting Information). With increasing DC to AC voltage ratio at constant maximum voltage, the intensity of SPL at the fundamental frequency (first harmonic, 10 kHz) increased, while the intensity of the second harmonic peak (20 kHz) decreased, which indicated an improvement of fidelity in the sound of stretchable EL loudspeakers (Figure S20, Supporting Information).

However, as the DC voltage increased above 280 V, SPL steeply decreased because of the insufficient AC drive voltage. At a fixed applied bias, SPL gradually increased with increasing frequency for the range of 1–25 kHz (Figure S21, Supporting Information). Moreover, at a constant electric field and frequency, SPL increased with decreasing thickness of the emission layer because of the concomitant increase in the concentration of the electric field on ZnS:Cu phosphors (Figure S22, Supporting Information). Variation of the distance between the microphone and loudspeaker between 2 and 30 cm showed that output sound pressure was well maintained over a long distance (Figure 4e), which was indicative of superior loudspeaker performance. The SPL‐distance relationship is known to follow the inverse square law, that is, SPL decreases by 6 dB upon the doubling of the distance from the sound source. However, this relationship holds for distances of greater than 1 m, as at shorter distances, near‐field effects result in a complex sound field close to the sound source.^[^ ^53^ ^]^ In our case, the invariability of loudspeaker sound performance with distance was ascribed to the fact that the measurement system was located in the near‐field region, that is, at a distance of <1 m. In contrast, a linear decrease in SPL with increasing distance between the loudspeaker and microphone is observed for thermoacoustic loudspeakers even in the near‐field region because of the rapid concomitant decrease of the temperature wave amplitude.^[^ ^43^, ^54^ ^]^ One of the potential issues of stretchable and bendable sound‐in‐display devices is the non‐uniform sound generation toward the listener at different off‐axis locations. Omnidirectional loudspeakers generate sound evenly in all directions, which is a significant advantage for the practical applications of sound‐in‐display devices. Our stretchable EL loudspeaker exhibited an omnidirectional sound‐generation performance (Figure 4f), that is, a sound wave with uniform SPL was generated irrespective of the detection angle, which was attributed to the isotropic vibration of the DEA film.

The stretchability of our loudspeaker was probed by measuring its output sound pressure upon increasing tensile strain up to 150% (Figure 4g), and the elongation‐induced sound performance change was found to be negligible because of the stable electrical connection provided by the stretchable AgNW electrodes. Loudspeaker stability and durability were probed by recording SPL during 5000 stretching cycles (Figure 4h), and the negligible SPL change was ascribed to the presence of highly robust and strain‐insensitive stretchable AgNW electrodes and the ZnS:Cu/PDMS emitting layer. The practical utility of sound‐in‐display devices based on our stretchable EL loudspeaker was demonstrated by playing Mozart's “Le Nozze di Figaro” in the dark anechoic chamber (Movie S1, Supporting Information). During this experiment, the stretchable EL loudspeaker emitted luminescence light synchronized with music, and the stable generation of sound from the highly stretchable loudspeaker resulted in high sound quality even when the loudspeaker was stretched and twisted (Movies S2 and S3, Supporting Information). Moreover, our loudspeaker showed negligible changes in sound quality and performance even after 5000 on–off cycles, and was therefore concluded to exhibit high long‐term stability (Figure 4i).

**Figure 5:**
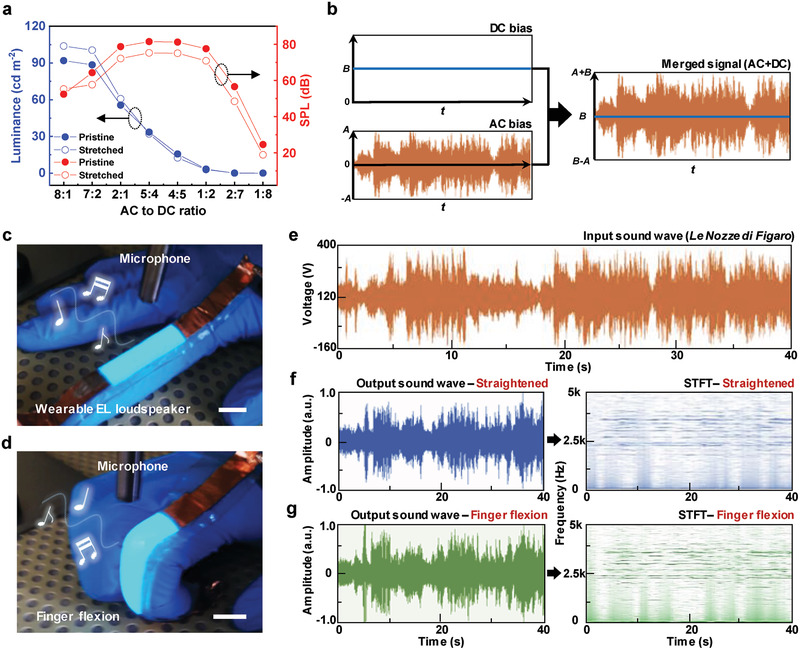
Stretchable sound‐in‐display electronics. a) Luminance and SPL as functions of the AC to DC voltage ratio (*V*_max_ = 360 V, frequency = 10 kHz) before and after the application of tensile strain (150%). b) Schematic diagram showing the waveform of AC‐DC combined signals used for music playing. c) Wearable EL loudspeaker attached to a gloved index finger during music playing. Music sound was recorded by a commercial microphone. Scale bar is 1.5 cm. d) Wearable EL loudspeaker during finger flexion. Scale bar is 1.5 cm. e) Input signal of the sound wave ("Le Nozze di Figaro"). f) Analyzed output sound wave (left) and its short‐time Fourier transform (right) in the straightened state and g) in the state of finger flexion.

As a proof of concept application, we fabricated wearable sound‐in‐display electronics using the stretchable EL loudspeaker, which offered a synesthetic stimulus with synchronous light and sound during music playing. The AC to DC applied voltage ratio was optimized to ensure the best light and sound‐generation performances. **Figure** **5**a shows the luminance and SPL of the stretchable EL loudspeaker as functions of the AC to DC ratio at constant combined maximum voltage (400 V) and frequency (10 kHz) before and after the application of tensile strain (150%). Whereas luminance continuously decreased with decreasing proportion of AC voltage because of the insufficiency of this voltage for phosphor excitation, SPL initially increased with increasing DC bias fraction because of the concomitant improvement of DEA actuation. However, overly high proportions of DC bias led to a decrease in SPL due to the lack of AC drive voltage. Besides, the stretchable EL loudspeaker showed negligible change in luminance and SPL during stretching, and was therefore concluded to be well suited for use in stretchable sound‐in‐display electronics. To demonstrate the ability of the EL loudspeaker to stably play music under stretching conditions, the input signal of the music sound wave was merged with the DC bias (Figure 5b). When the wearable EL loudspeaker was attached to a human finger in the straightened state (Figure 5c), synchronous light and sound were produced along with the input sound wave signal. Thus, the EL loudspeaker provided an intuitive perception of auditory‐visual stimuli by visualizing the sound wave using emitted light. The loudspeaker could be stably operated even during finger flexion because of its high stretchability (Figure 5d). Figure 5e shows the input sound signal ("Le Nozze di Figaro") applied to the wearable EL loudspeaker. Here, the offset DC bias could be controlled to improve sound quality by enhancing the vibration of the DEA matrix during music playing. Figure 5f,g show the output sound waveforms and short‐time Fourier transform (STFT) spectrograms of the music sound played by the wearable EL loudspeaker before and after finger flexion, respectively, revealing that this flexion did not significantly affect the output sound waves and STFT spectrograms and thus indicating the maintenance of sound quality, excellent stretchability, and reliability of our device. When “Le Nozze di Figaro” was played during repeated finger bending, no noticeable change in sound and emitted light were observed (Movie S4, Supporting Information), which indicated the high performance stability of our wearable EL loudspeaker.

## 3 Conclusion

In summary, synchronized generation of color and sound in a single sound‐in‐display device was realized using a stretchable EL loudspeaker comprising ZnS:Cu EL phosphors dispersed in a stretchable PDMS DEA layer sandwiched between stretchable AgNW electrodes. The loudspeaker simultaneously emitted light by the EL of ZnS:Cu phosphors and generated sound waves by the vibration of the PDMS DEA under the same AC bias input, allowing the synesthetic perception of both color and sound. The intensity of light emission and output sound generation could be easily controlled by the frequency and amplitude of the applied AC bias. Importantly, the stable electrical connection provided by strain‐insensitive stretchable AgNW electrodes resulted in highly robust and reliable EL and sound emission performances even under significant tensile strains of up to 150% and 5000 stretch‐release cycles. As a proof of concept application, our loudspeaker was integrated in a stretchable sound‐in‐display device that could be worn on a human finger and provided a synesthetic vision and hearing stimulus with synchronous light and sound generation even when significantly stretched during finger flexion. Thus, the presented loudspeaker with the capability of synchronized sound generation and visual display in a single sound‐in‐display device is a robust platform for the further development of human–machine interfaces in the visualization of auditory systems and IoT devices.

## 4 Experimental Section

4.1

4.1.1

##### 4.1.1.1 Fabrication of Strain‐Insensitive Silver Nanowires Stretchable Electrodes

For elastic substrate preparation, a PDMS base (Sylgard 184, Dow Corning) and PDMS curing agent were thoroughly mixed in a 10:1 mass ratio, and the mixture was degassed in a vacuum desiccator for 30 min to remove bubbles and then evenly poured on a Petri dish. For the partial curing of PDMS, the Petri dish with PDMS was heated in an oven for 20 min at 80 °C. Prior to being coated with the AgNW dispersion, partially cured PDMS was pre‐stretched using an in‐house‐made stretching stage and pre‐treated with O_2_ plasma (18 W, 5 min). The dispersion of AgNWs (average length = 22 µm, diameter = 21 nm; Flexiowire 2020, Flexio Co., Ltd.) was spun onto the partially cured and pre‐stretched PDMS at a spin rate of 2000 rpm for 120 s, and the coated substrate was released and thermally cured at 80 °C for 2 h. Finally, Cu tapes and silver paste were applied to the edges of stretchable electrodes.

##### 4.1.1.2 Fabrication of Stretchable Electroluminescent Loudspeaker

The ZnS:Cu/PDMS composite was prepared by thoroughly mixing ZnS:Cu microparticle powder (Shanghai KPT Co.) with liquid PDMS in a 2:1 mass ratio using a planetary mixer (ARE‐310, Thinky Corporation) at a mixing rate of 2000 rpm for 15 min. The thus obtained composite was spun onto the bottom stretchable electrode at a spin rate of 2000 rpm for 120 s, and the top electrode was then attached at the top of the composite layer. Finally, the assembled device was thermally cured at 80 °C for 2 h.

##### 4.1.1.3 Characterization

The surface morphology of strain‐insensitive stretchable electrodes was examined by optical microscopy (PSM‐1000, Olympus) and field‐emission SEM (Hitachi S4800, operating voltage = 10 kV). The latter technique was also used to probe the surface morphology of ZnS:Cu phosphors. Optical transmittance was determined using ultraviolet‐visible spectroscopy (Carry 5000, Agilent), and electrical resistance was measured by a multimeter (3201, Müller). Stretching tests were performed using an in‐house‐made stretching apparatus. The fabricated EL loudspeaker device was connected to the Cu tapes at the edges of stretchable electrodes for the application of electric field. A function generator (AFG 3011C, Tektronix) connected with a power amplifier (T‐700H, ElbaTech) was used to apply a square alternating voltage to the stretchable EL loudspeaker through the Cu tape electrodes. For music playing, input sound waves of a sinusoidal waveform and an offset bias voltage were applied using LabVIEW software. Luminescence spectra of stretchable EL loudspeaker devices were recorded by a spectroradiometer (PR‐655, PhotoResearch, Inc.). Luminance was measured in a dark chamber. A dynamic signal analyzer (National Instruments Corp.) integrated with a commercial microphone (40PH, GRAS) was used to capture the sound emitted by the stretchable EL loudspeaker. Sound tests were performed in an anechoic chamber with a cut‐off frequency of 500 Hz and a sound transmission class of >30 dB (SMB‐80, IDS‐technology).

## Conflict of Interest

The authors declare no conflict of interest.

## Supporting information

Supporting Information

Supporting Information

Supporting Information

Supporting Information

Supporting Information
