# Supplementary material for: Highly Stretchable Sound‐in‐Display Electronics Based on Strain‐Insensitive Metallic Nanonetworks
Source: Adv Sci (Weinh). 2020 Nov 23;8(1):2001647. doi: 10.1002/advs.202001647 (PMC7788580; doi:10.1002/advs.202001647)
Supplement: Supplementary file 1 — Supporting Information [file ADVS-8-2001647-s001.pdf]

© 2020 Wiley-VCH GmbH

ADVANCED SCIENCE
Open Access

Supporting Information

for *Adv. Sci.,* DOI: 10.1002/advs.202001647

Highly Stretchable Sound-in-Display Electronics Based on Strain-Insensitive Metallic Nanonetworks

*Seungse Cho, Dong-hee Kang, Hyejin Lee, Minsoo P. Kim, Saewon Kang, Ravi Shanker, and Hyunhyub Ko**

Supporting Information

Highly Stretchable Sound-in-Display Electronics Based on Strain-Insensitive Metallic Nanonetworks

*Seungse Cho, Dong-hee Kang, Hyejin Lee, Minsoo P. Kim, Saewon Kang, Ravi Shanker, and Hyunhyub Ko**

Supplementary Note 1. **Fitting of the relationship between EL intensity and applied voltage bias at a certain frequency**

To fit the relationship between EL intensity and applied voltage bias at a certain frequency (Equation 1), two experimental luminance values obtained at applied voltages of 20 and 180 V were used to determine the values of L_0 and β, which were calculated as 66.82 and 40.01 (100 Hz), 520.2 and 40.19 (1 kHz), 1457.3 and 40.2 (5 kHz), 1912.5 and 40.19 (10 kHz), 2428.7 and 40.18 (25 kHz), and 2637.7 and 40.18 (50 kHz), respectively. Luminance values (L) were calculated according to the determined values of L_0, β, and applied voltages between 10 and 180 V.

Supplementary Note 2. Harmonic distortion generated by the stretchable EL loudspeaker

The DC-AC combined voltage (V) applied to the DEA-based loudspeaker can be expressed as $V = B + A\sin(\omega t)$, where B is the DC bias and A is the AC drive voltage. As indicated in Equation 2, sound performance is related to film strain and is proportional to the square of applied voltage, which is given by $V^2 = B^2 + 2BA\sin(\omega t) + A^2\sin^2(\omega t)$. The above relationship can be re-written as $V^2 = B^2 + 2BA\sin(\omega t) + 0.5A^2[1 - \cos(2\omega t)]$, where the $\cos(2\omega t)$ term implies the generation of second harmonics with doubled frequency. The harmonic distortion primarily depends on the amplitude of AC drive voltage (A) compared to the DC voltage bias (B). Therefore, with increasing DC to AC voltage ratio, the amplitude of the harmonic peak decreases, which results in better sound performance and higher fidelity.

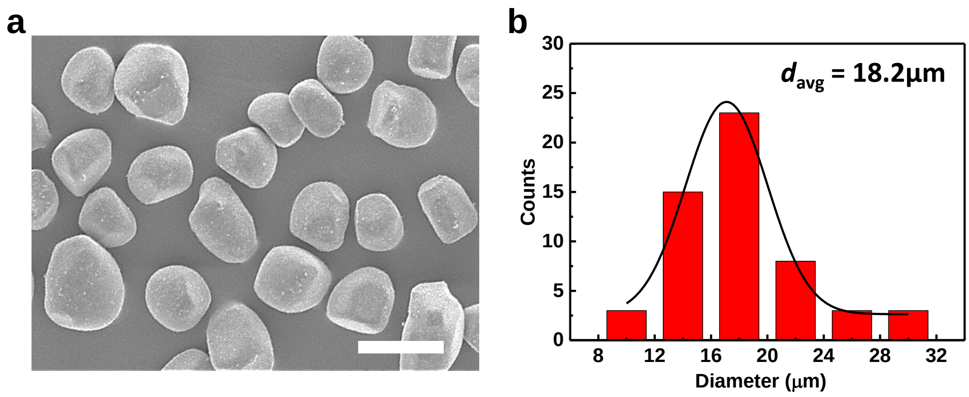

Figure S1. (a) Representative SEM image. Scale bar is 25 µm. (b) size distribution of ZnS:Cu phosphor particles.

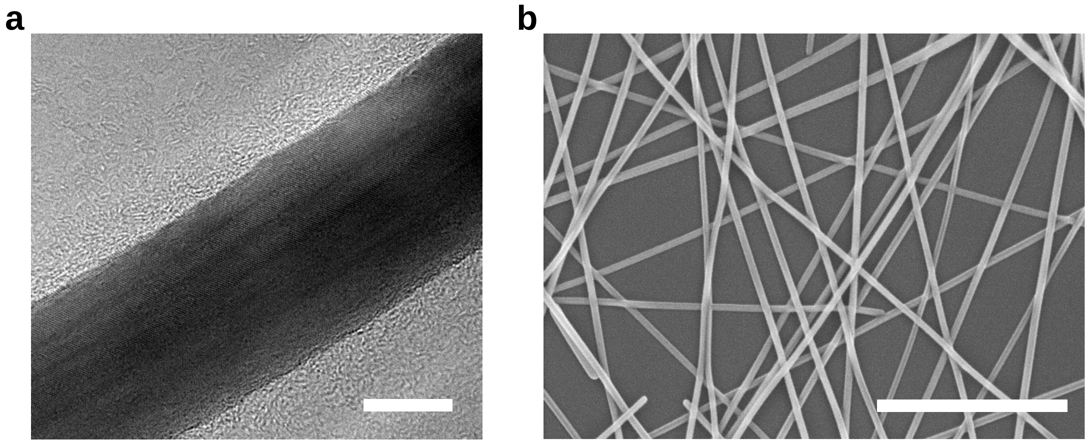

Figure S2. (a) TEM image of a single AgNW. Scale bar is 10 nm. (b) SEM image of the AgNW network. Scale bar is 1 µm.

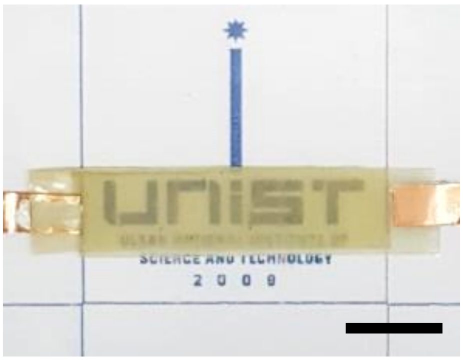

Figure S3. Photograph of the fabricated stretchable EL loudspeaker placed on the background UNIST logo. Scale bar is 1.5 cm.

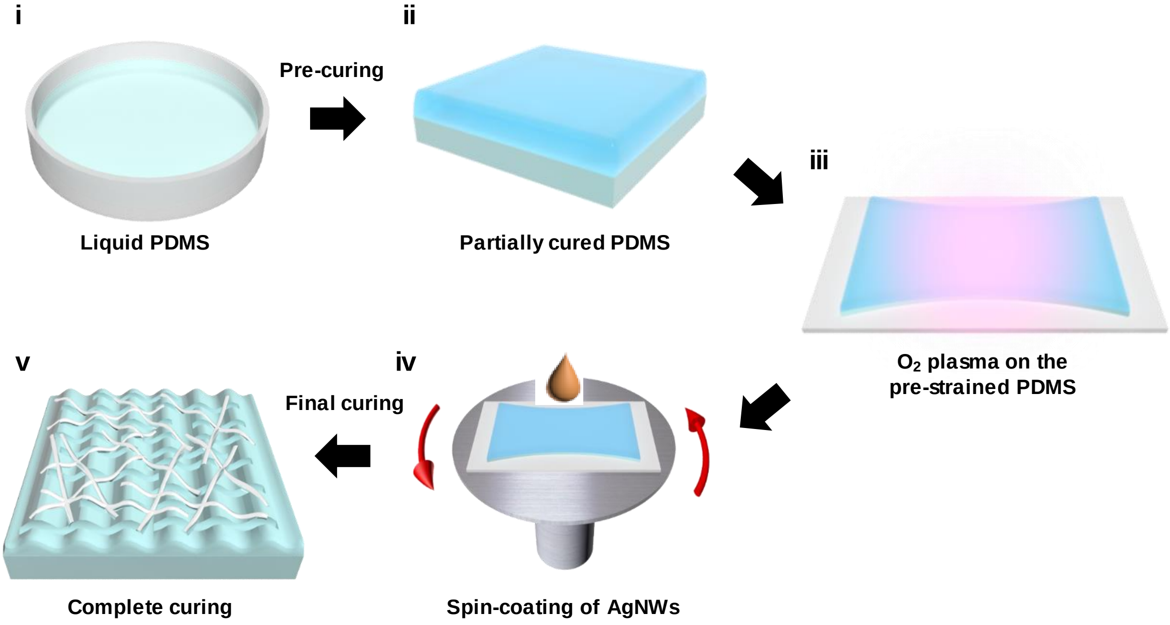

Figure S4. Schematic fabrication procedure of stretchable electrodes comprising strain-insensitive AgNWs. The liquid PDMS prepolymer (i) is partially cured. The partially cured PDMS (ii) is pre-strained, treated with O_2 plasma (iii), coated with AgNWs (iv), and finally released and cured completely (v).

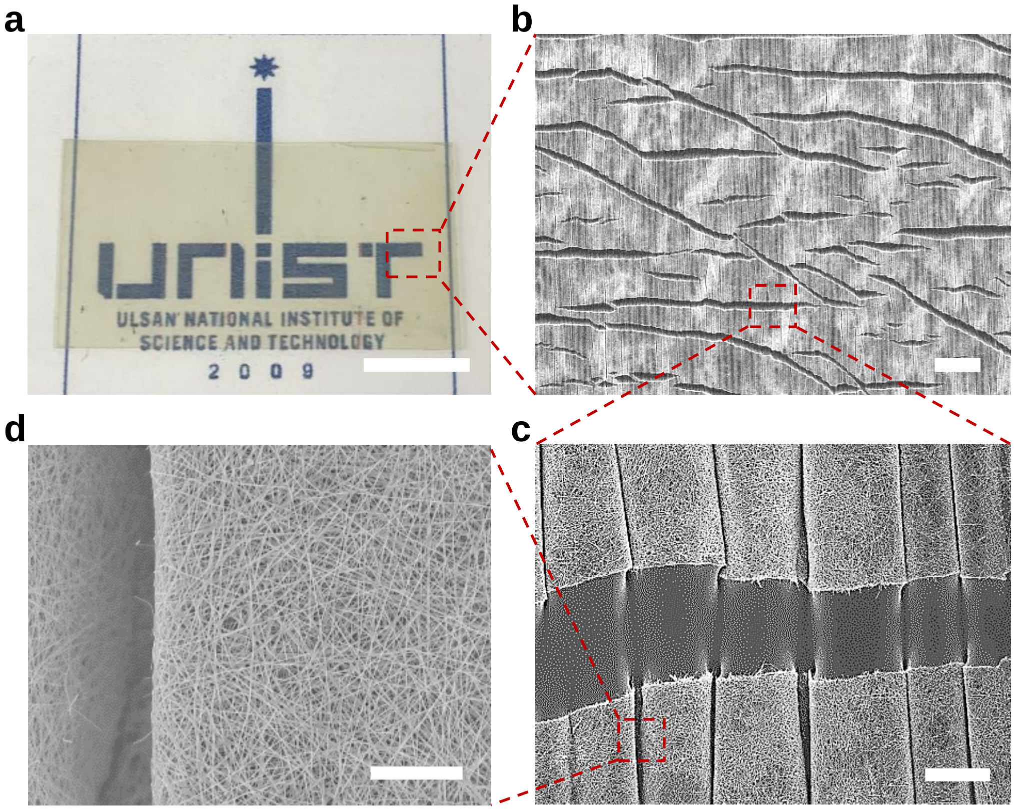

Figure S5. Observation of strain-insensitive stretchable AgNW electrodes. (a) Photograph and (b) representative SEM image of a stretchable and transparent AgNW electrode. Scale bars are 1.5 cm and 100 μm, respectively. (c, d) Magnified SEM images of the stretchable AgNW electrode. Scale bars are 10 and 3 μm, respectively.

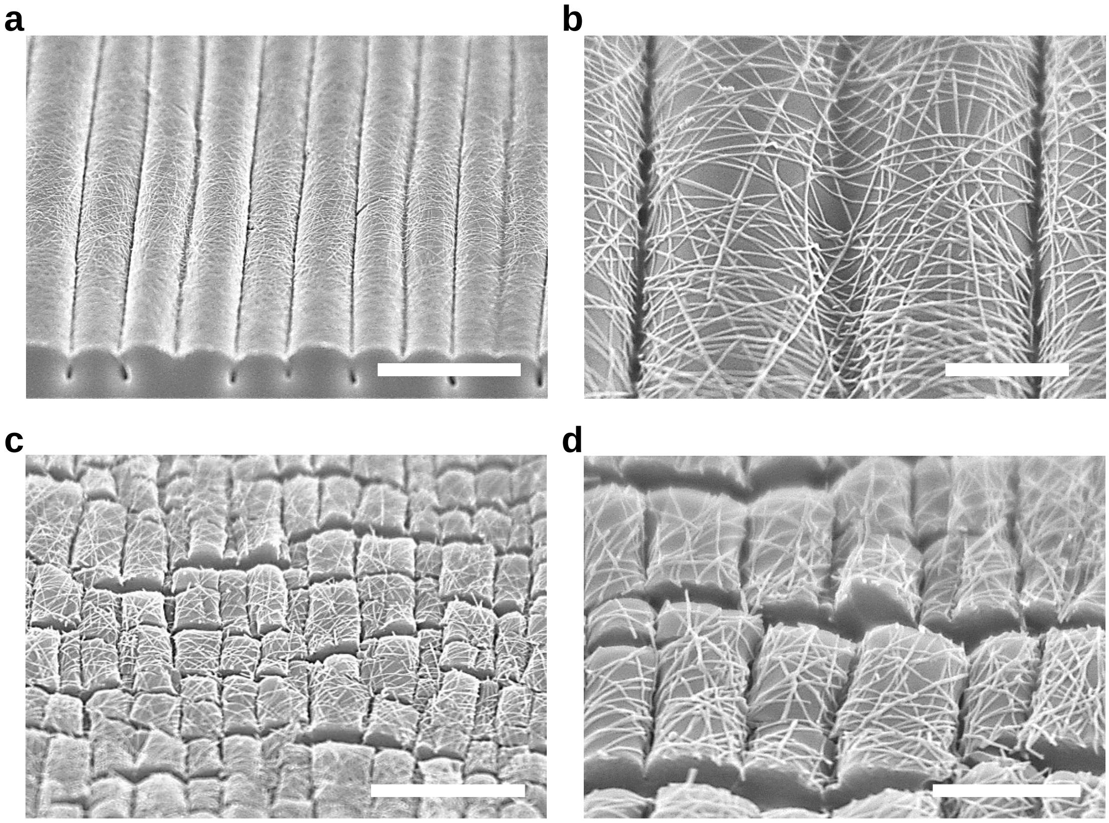

Figure S6. Comparison between stretchable AgNW electrodes fabricated using partially cured and fully cured PDMS. (a, b) SEM images of stretchable AgNW electrodes fabricated using partially cured PDMS. Scale bars are 10 and 2 µm, respectively. (c, d) SEM images of stretchable AgNW electrodes fabricated using fully cured PDMS. Scale bars are 5 and 2 µm, respectively.

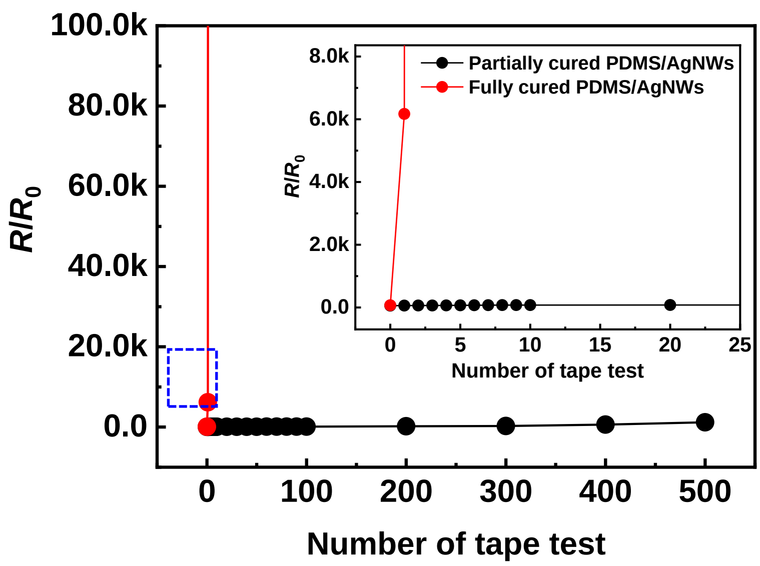

Figure S7. Effects of taping tests on the relative resistance (R/R_0) of stretchable AgNW electrodes fabricated using partially cured and fully cured PDMS. In contrast to the electrodes based on partially cured PDMS, those based on fully cured PDMS showed a dramatic increase in resistance after two tests.

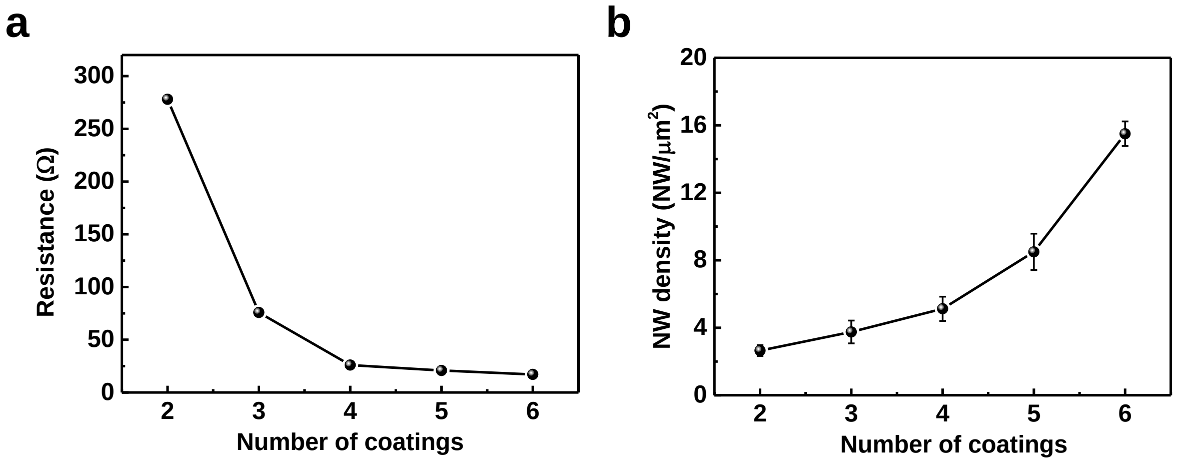

Figure S8. Effect of the number of coatings on (a) the electrical resistance of stretchable AgNW electrodes and (b) AgNW density.

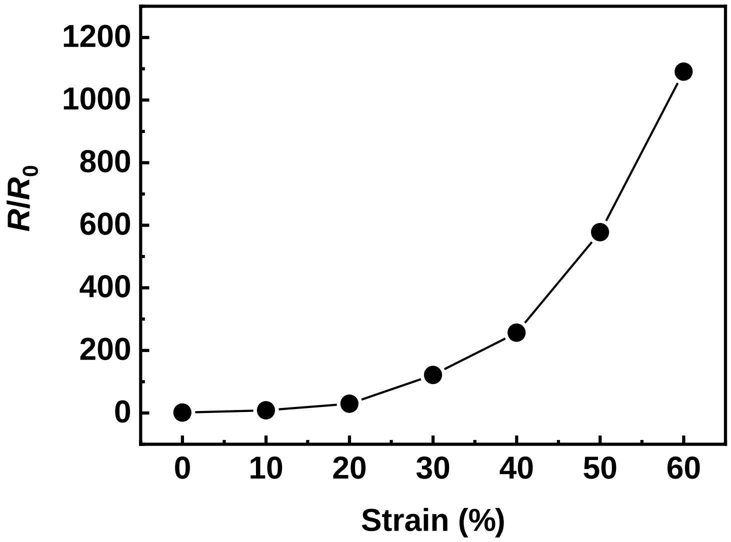

Figure S9. Effect of strain on the resistance of stretchable AgNW electrodes fabricated without pre-straining.

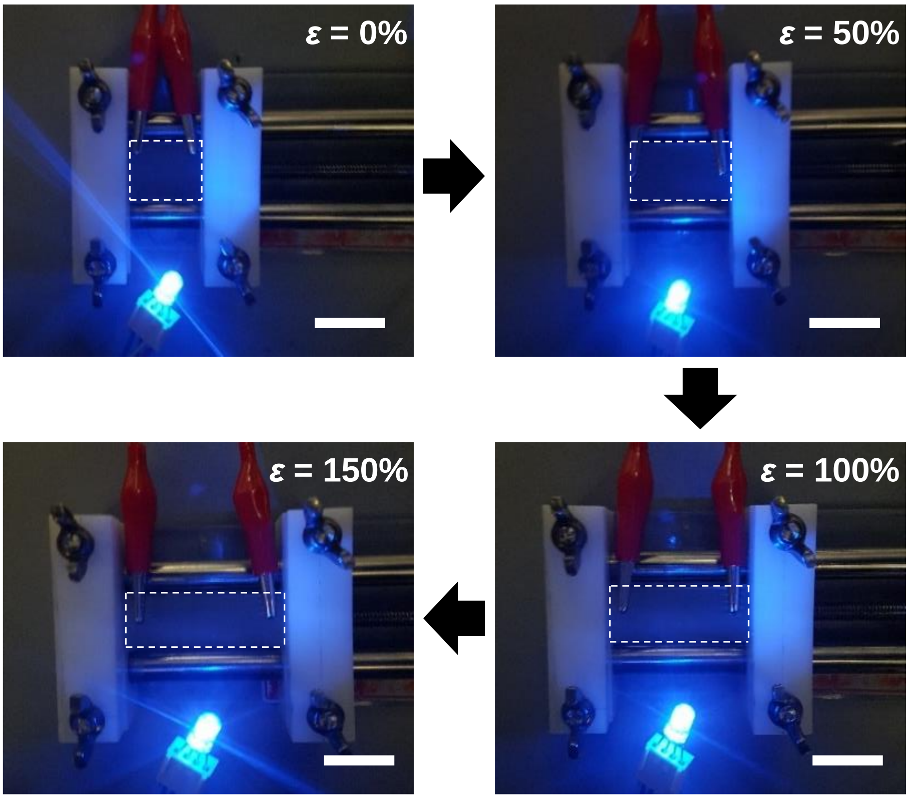

Figure S10. Photographs showing the behavior of LED circuits connected to strain-insensitive stretchable AgNW electrodes upon the increase of tensile strain up to 150%. All scale bars equal 2 cm.

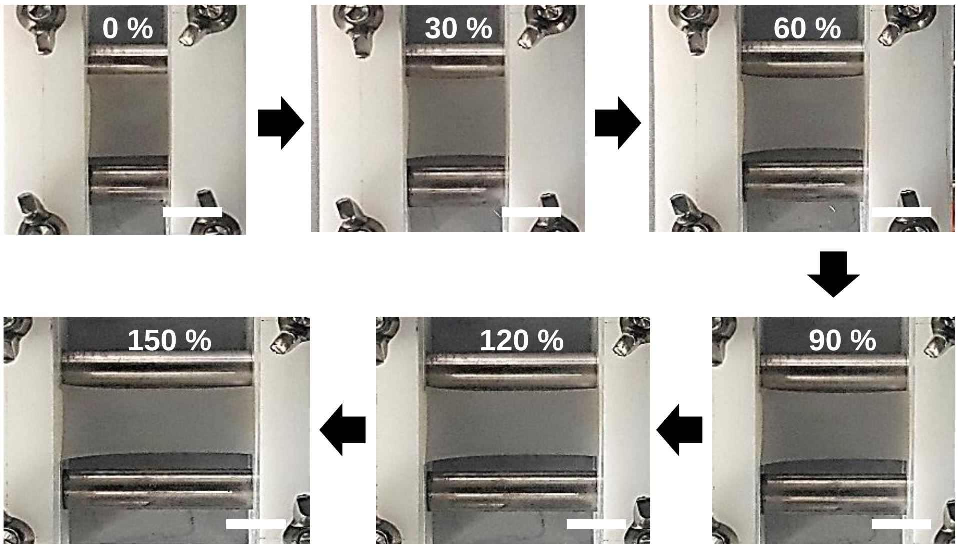

Figure S11. Photographs of a stretchable electrode during stretching to 150%. All scale bars equal 1 cm.

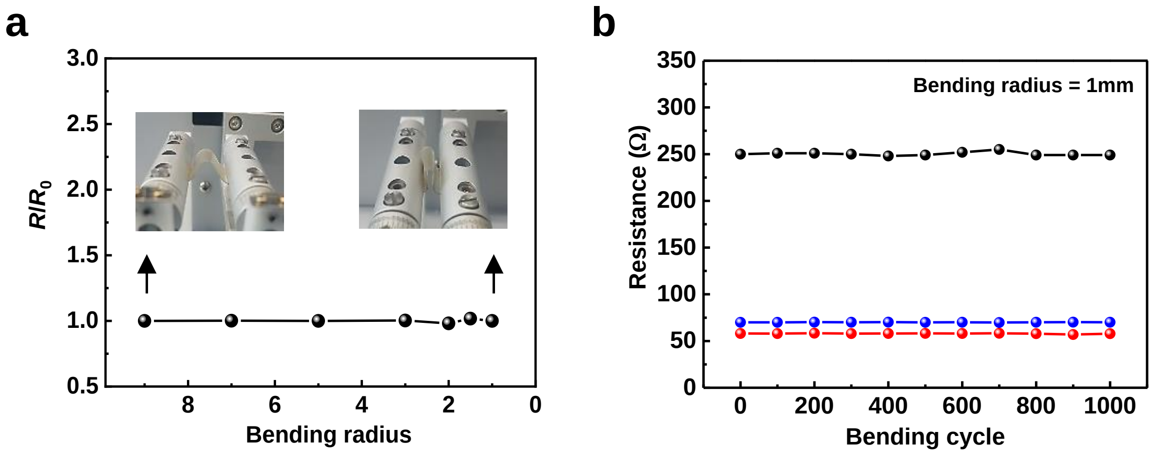

Figure S12. Mechanical stability of strain-insensitive stretchable AgNW electrodes. (a) Effect of bending radius on electrode resistance. (b) Electrode resistance change during 1000 bending cycles at a bending radius of 1 mm.

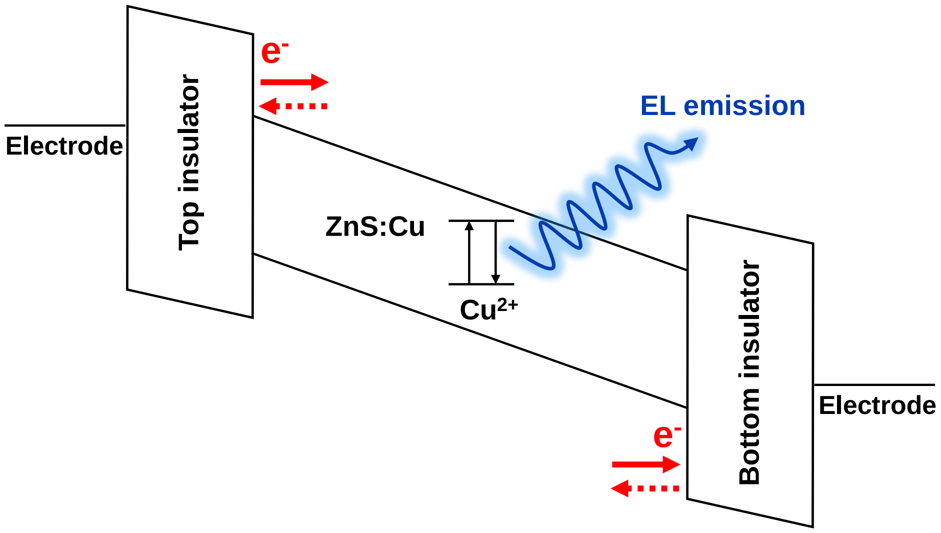

Figure S13. Band structure diagram showing the ACEL mechanism for the stretchable EL loudspeaker. Emission originates from the impact excitation of Cu^{2+} in ZnS:Cu phosphors.

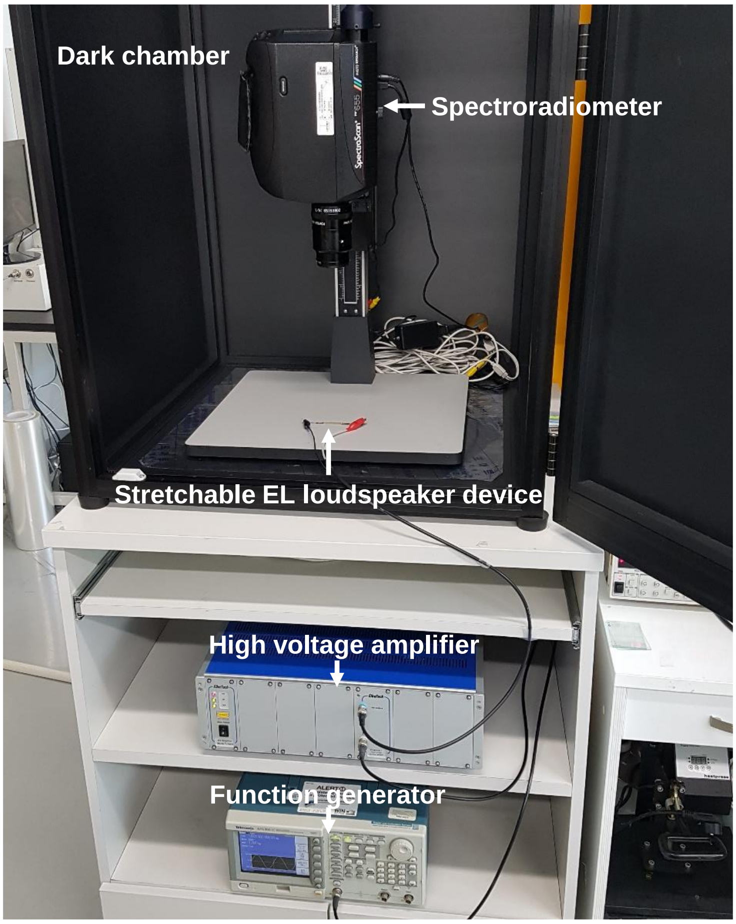

Figure S14. Photograph of the setup used to measure EL by a spectroradiometer. The applied AC voltage from the function generator was amplified by a high-voltage amplifier.

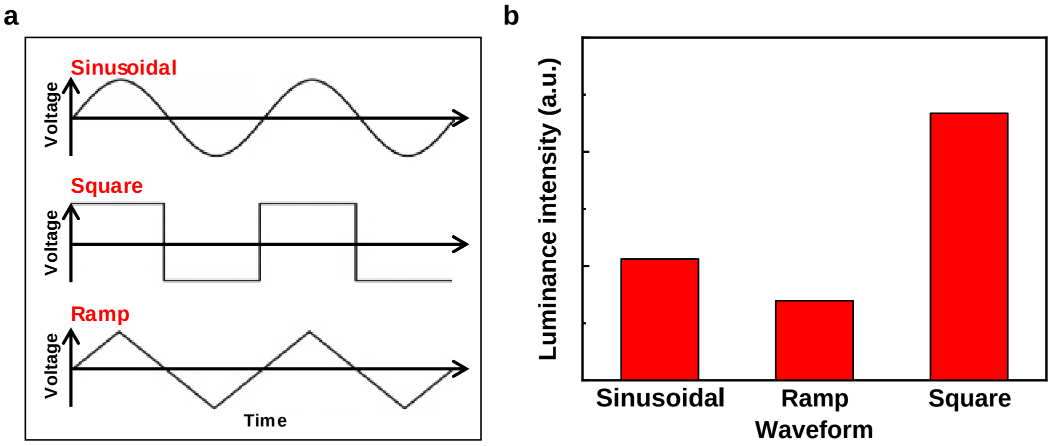

Figure S15. (a) Sinusoidal, square, and ramp waves. (b) Luminance intensity obtained for various excitation waveforms at 100 V and 10 kHz.

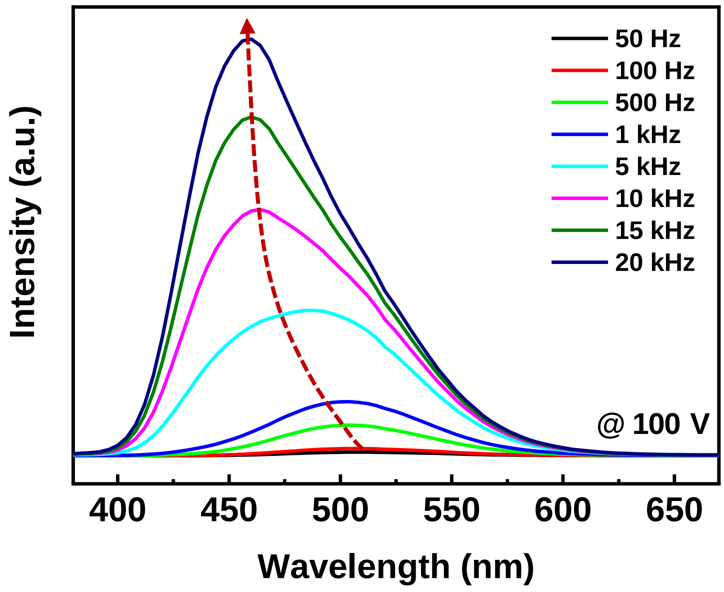

Figure S16. EL spectra as a function of applied frequency (50 Hz to 20 kHz) at a fixed applied voltage of 100 V.

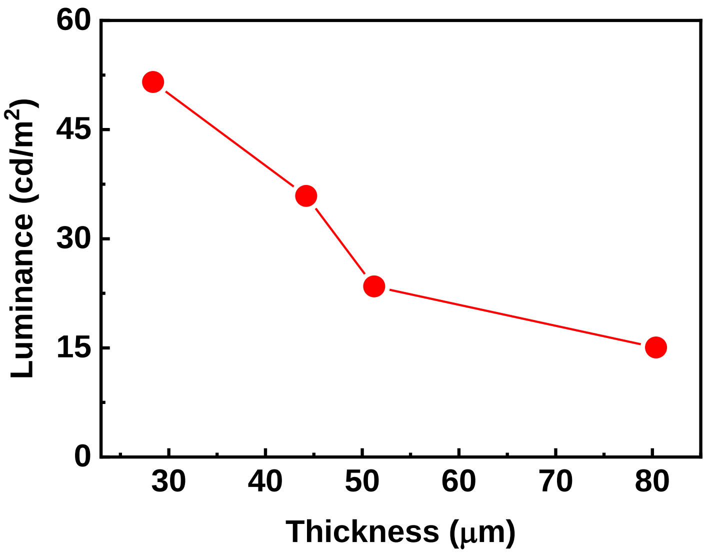

Figure S17. Luminance of stretchable EL loudspeaker as a function of emission layer thickness (100 V, 10 kHz).

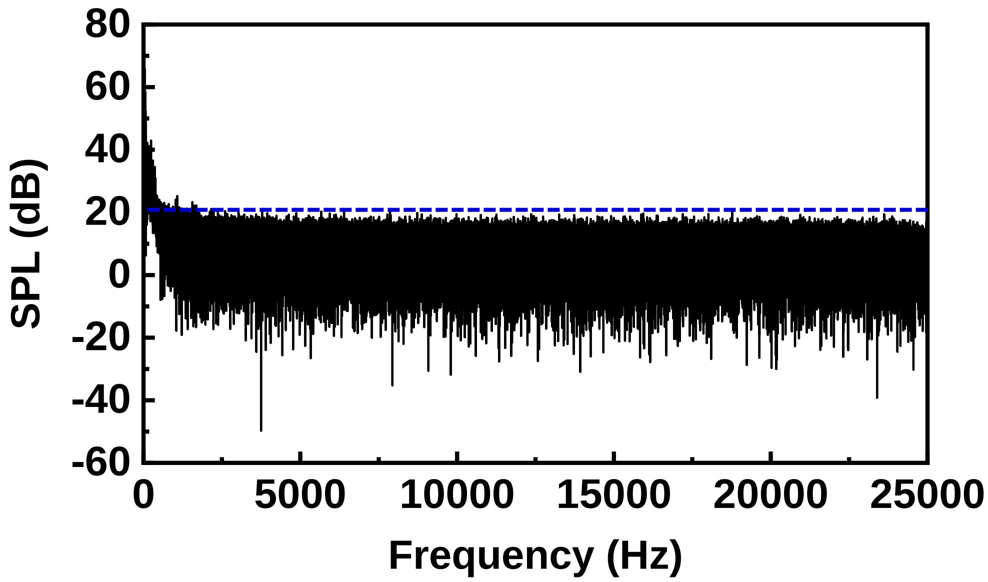

Figure S18. Power spectrum of background noise in the anechoic room.

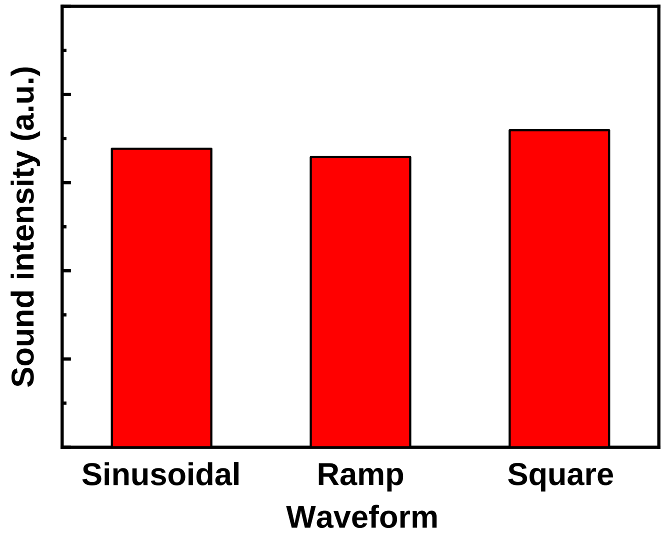

Figure S19. Sound intensity under varying excitation waveforms (100 V, 10 kHz).

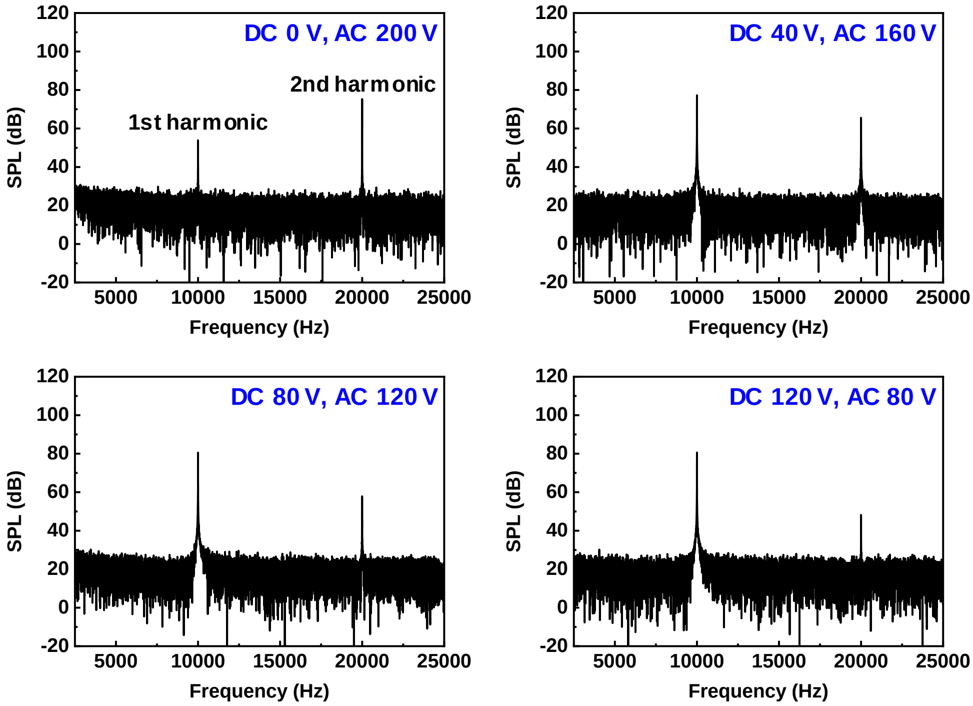

Figure S20. Sound performance at different DC to AC bias ratios (V_{max} = 200 V, 10 kHz).

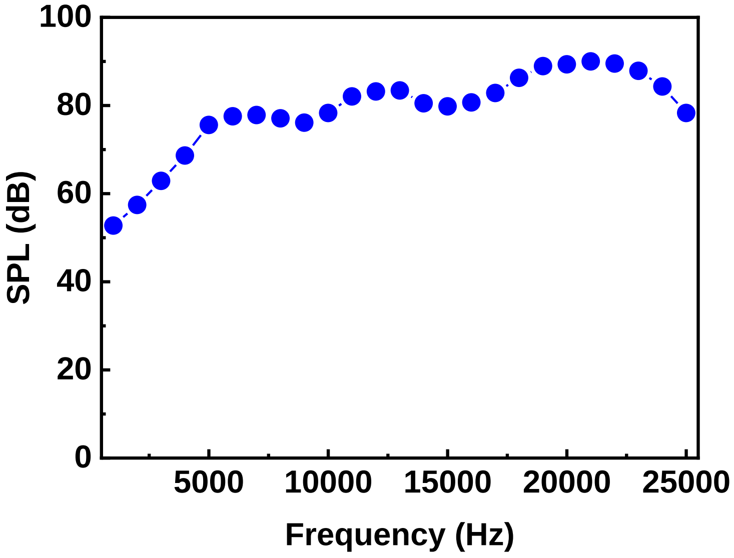

Figure S21. Effect of frequency on the SPL of the stretchable EL loudspeaker.

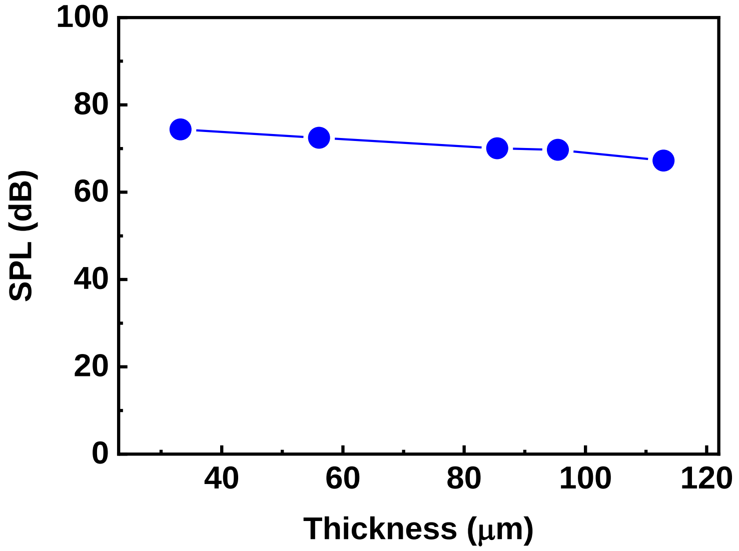

Figure S22. Effect of emission layer thickness on the SPL of the stretchable EL loudspeaker (100 V, 10 kHz).

Table S1. Summary of recently reported works related to the light emission or sound generation.

Materials for			Stretchability	Luminance	SPL	Ref
Electrodes	EL	Sound (Principle)				
AgNW/PDMS	ZnS	-	100%	> 12 cd/m^2 (120V, 1 kHz)	-	[1]
AgNW/PDMS	ZnS	-	80%	> 5 cd/m^2 (120 V, 0.4 kHz)	-	[2]
Ionic gel/ VHB tape	ZnS	-	1500% (Area)	> 2 cd/m^2 (900 V, 1 kHz)	-	[3]
AgNW/PDMS	ZnS/QDs	-	60%	~ 1 cd/m^2 (120 V, 1 kHz)	-	[4]
Polar solvent /Ag flake/PET	ZnS	-	-	~ 40 cd/m^2 (120 V, 1 kHz)	-	[5]
AgNW/PET	ZnS	-	-	~ 18 cd/m^2 (120 V, 1 kHz)	-	[6]
CNT	-	CNT (Thermoacoustic)	100%	-	> 100 dB (50 V, 10 kHz)	[7]
Galinstan	-	Galinstan/Nd (Vibration)	50%	-	> 50 dB (1 V, 10 kHz)	[8]
Sputtered Ag	-	PP/silicate particle (Ferroelectret)	-	-	> 90 dB (300 V, 10 kHz)	[9]
Graphene	-	Graphene (Thermoacoustic)	-	-	> 45 dB (0.25 W, 10 kHz)	[10]
PEDOT:PSS	SE-ZnS	PVDF-TrFE (Piezoelectric)	-	~ 35 cd/m^2 (100 V, 1 kHz)	> 60 dB (120 V, 10 kHz)	[11]
**Wrinkled AgNW/PDMS**	**ZnS**	**PDMS (Vibration)**	**150%**	**~ 15.1 cd/m^2 (120 V, 1 kHz)**	**~ 83.3 dB (120 V, 10 kHz)**	**This work**

References

[1] J. Wang, C. Yan, K. J. Chee, P. S. Lee, *Adv. Mater.* **2015**, *27*, 2876.

[2] F. Stauffer, K. Tybrandt, *Adv. Mater.* **2016**, *28*, 7200.

[3] C. H. Yang, B. Chen, J. Zhou, Y. M. Chen, Z. Suo, *Adv. Mater.* **2016**, *28*, 4480.

[4] S. M. Jeong, S. Song, H. Kim, S.-H. Baek, J. S. Kwak, *RSC Adv.* **2017**, *7*, 8816.

[5] X. Xu, D. Hu, L. Yan, S. Fang, C. Shen, Y.-L. Loo, Y. Lin, C. S. Haines, N. Li, A. A. Zakhidov, H. Meng, R. H. Baughman, W. Huang, *Adv. Mater.* **2017**, *29*, 1703552.

[6] R. Shanker, S. Cho, A. Choe, M. P. Kim, Z. Khan, S. Kang, H. Ko, *Adv. Funct. Mater.* **2019**, *29*, 1904377.

[7] L. Xiao, Z. Chen, C. Feng, L. Liu, Z.-Q. Bai, Y. Wang, L. Qian, Y. Zhang, Q. Li, K. Jiang, S. Fan, *Nano Lett.* **2008**, *8*, 4539.

[8] S. W. Jin, J. Park, S. Y. Hong, H. Park, Y. R. Jeong, J. Park, S.-S. Lee, J. S. Ha, *Sci. Rep.* **2015**, *5*, 11695.

[9] W. Li, D. Torres, R. Dóaz, Zhengjun Wang, C. Wu, C. Wang, Z. L. Wang, N. Sepúlveda, *Nat. Commun.* **2017**, *8*, 15310.

[10] J. W. Suk, K. Kirk, Y. Hao, N. A. Hall, R. S. Ruoff, *Adv. Mater.* **2012**, *24*, 6342.

[11] J. S. Kim, S. H. Cho, K. L. Kim, G. Kim, S. W. Lee, E. H. Kim, B. Jeong, I. Hwang, H. Han, W. Shim, T.-W. Lee, C. Park, *Nano Energy* **2019**, *59*, 773.
